# Estrogen-related receptor alpha (ERRα) is a key regulator of intestinal homeostasis and protects against colitis

**DOI:** 10.1038/s41598-021-94499-5

**Published:** 2021-07-23

**Authors:** Allan Tran, Charlotte Scholtes, Mario Songane, Claudia Champagne, Luc Galarneau, Marie-Pier Levasseur, Nassima Fodil, Catherine Rosa Dufour, Vincent Giguère, Maya Saleh

**Affiliations:** 1 Department of Microbiology and Immunology, McGill University, Montreal, QC H3A 2B4 Canada; 2 Goodman Cancer Research Centre, McGill University, Montreal, QC H3A 2B4 Canada; 3 Department of Medicine, McGill University, Montreal, QC H3G 0B1 Canada; 4 Cedars Cancer Centre, Medical Physics, McGill University Health Centre, Montreal, H4A 3J1 Canada; 5 Department of Biochemistry, McGill University, Montreal, QC H3A 2B4 Canada; 6 Department of Life Sciences and Health, CNRS, ImmunoConcEpT, UMR 5164, The University of Bordeaux, 33000 Bordeaux, France

**Keywords:** Mucosal immunology, Gastroenterology

## Abstract

The estrogen-related receptor alpha (ERRα) is a primary regulator of mitochondrial energy metabolism, function and dynamics, and has been implicated in autophagy and immune regulation. ERRα is abundantly expressed in the intestine and in cells of the immune system. However, its role in inflammatory bowel disease (IBD) remains unknown. Here, we report a protective role of ERRα in the intestine. We found that mice deficient in ERRα were susceptible to experimental colitis, exhibiting increased colon inflammation and tissue damage. This phenotype was mediated by impaired compensatory proliferation of intestinal epithelial cells (IEC) following injury, enhanced IEC apoptosis and necrosis and reduced mucus-producing goblet cell counts. Longitudinal analysis of the microbiota demonstrated that loss of ERRα lead to a reduction in microbiome α-diversity and depletion of healthy gut bacterial constituents. Mechanistically, ERRα mediated its protective effects by acting within the radio-resistant compartment of the intestine. It promoted disease tolerance through transcriptional control of key genes involved in intestinal tissue homeostasis and repair. These findings provide new insights on the role of ERRα in the gut and extends our current knowledge of nuclear receptors implicated in IBD.

## Introduction

Inflammatory bowel diseases (IBD) are a group of chronic idiopathic inflammatory disorders of the gastrointestinal tract characterized by alternating phases of relapse and remission. The two main forms of IBD are Crohn’s disease (CD) and ulcerative colitis (UC). While the etiology of IBD remains elusive, it is thought to arise from an inappropriate immune response to the intestinal microbiota in genetically susceptible individuals. The monolayer of the intestinal epithelium acts as a defensive barrier that ensures the segregation of luminal contents from the mucosal immune system. Defects in epithelial integrity or function contribute to dysregulated mucosal immunity, associated with perturbations in the intestinal microbiome. IBD is characterized by loss of microbiota bacterial diversity, expansion of Proteobacteria (e.g. increase in *Escherichia* spp.) and depletion of firmicutes (e.g. loss of *Faecalibacterium prausnitzii*)^1^. Despite inter-individual variations, IBD-associated gut dysbiosis is accompanied by consistent metabolic alterations, notably a reduction in short chain fatty acids (SCFAs), secondary bile acids and acylcarnitines^2^, which link microbiota dysbiosis to host metabolism, particularly fatty acid oxidation (FAO) in the mitochondria.

A fine-tuned regulation of the turnover of the gut epithelium and maintenance of immunological tolerance are tightly regulated by mitochondrial function. Mitochondrial metabolism regulates intestinal homeostasis by orchestrating intestinal epithelial cells (IEC) regeneration and differentiation^3^. Additionally, the mitochondria serve as structural and functional hubs for innate immunity signaling^4^. Quality control of the mitochondria and energy metabolism are contingent on mitochondrial biogenesis and dynamics through fission and fusion, the mitochondrial unfolded protein response (mt-UPR), and the autophagy-dependent degradation of damaged mitochondria, known as mitophagy^5^. Defects in these pathways are linked to IBD in genetic-association and functional studies^6, 7^. Mitochondrial abnormalities, such as a swollen structure and irregular cristae have been observed in the enterocytes of IBD patients, and higher mitochondrial DNA levels were found in their plasma and feces^8^. Moreover, IBD patients have an energy deficit, with lower ATP levels in their intestinal mucosa^9^ and a dysfunctional mitochondrial respiratory chain complex activity^10, 11^. Similarly, mice with colitis exhibit downregulated mitochondrial biogenesis, impaired mitochondrial function and structure, and oxidative stress^12^. Defects in autophagy and mitophagy are also associated with IBD, by impacting IEC survival and function, anti-microbial peptide production, bacterial handling, reactive oxygen species (ROS) levels and immune responses to the microbiota.

Several nuclear receptors (NRs) regulate gut physiology and counter intestinal inflammation^13^, suggesting that the use of their ligands/agonists could benefit IBD patients. Concordantly, the Peroxisome proliferator-activated receptor (PPAR)γ agonist, rosiglitazone, has shown some efficacy in the treatment of mild to moderately active UC in a phase 2 clinical trial^14^. The estrogen-related receptor α (ERRα; NR3B1), a member of the orphan NR family, is a central regulator of mitochondrial energy metabolism, mitochondrial biogenesis and dynamics^15^. ERRα is expressed ubiquitously in all cells and tissues^16^, particularly in tissues with high oxidative capacity including the intestine, and acts at the intersection of cellular metabolism, oncogenesis, and immunity^17–21^. ERRα governs all aspects of mitochondrial function by regulating the expression of nuclear-encoded mitochondrial proteins^22^. Notably, it is required for the transport of acylcarnitines into the mitochondrial matrix for FAO, through transcriptional regulation of carnitine/acylcarnitine translocase (CACT)^23^. ERRα also regulates autophagy and mitophagy^24, 25^. The transcriptional activity of ERRα is dependent on the co-activators PGC-1α and PGC-1β^26–29^. PGC-1α, in particular, controls IEC fate by instructing a glycolytic-to-oxidative gradient along the crypt-villus axis^30^. Importantly, its levels are reduced in IBD and in experimental models of colitis, and its IEC-specific deletion in mice (PGC-1α Δ^IEC^) results in increased susceptibility to experimental colitis induced by dextran sodium sulfate (DSS)^12^.

Taken its role in energy metabolism, autophagy and immunity, and its close interaction with PGC-1α, we hypothesized that ERRα is critical in intestinal homeostasis. Here, we show that ERRα knockout mice (*Esrra*^−/−^) phenocopied PGC-1α deficiency in colitis, exhibiting a heightened disease severity, increased intestinal inflammation, loss of goblet cells and microbiota dysbiosis. Our study reveals an important role for ERRα in intestinal homeostasis, highlighting its potential as a therapeutic target in IBD.

## Results

### ***Esrra***^−/−^ mice exhibited heightened susceptibility to experimental colitis associated with impaired intestinal epithelial regeneration and increased cell death

**Figure 1:**
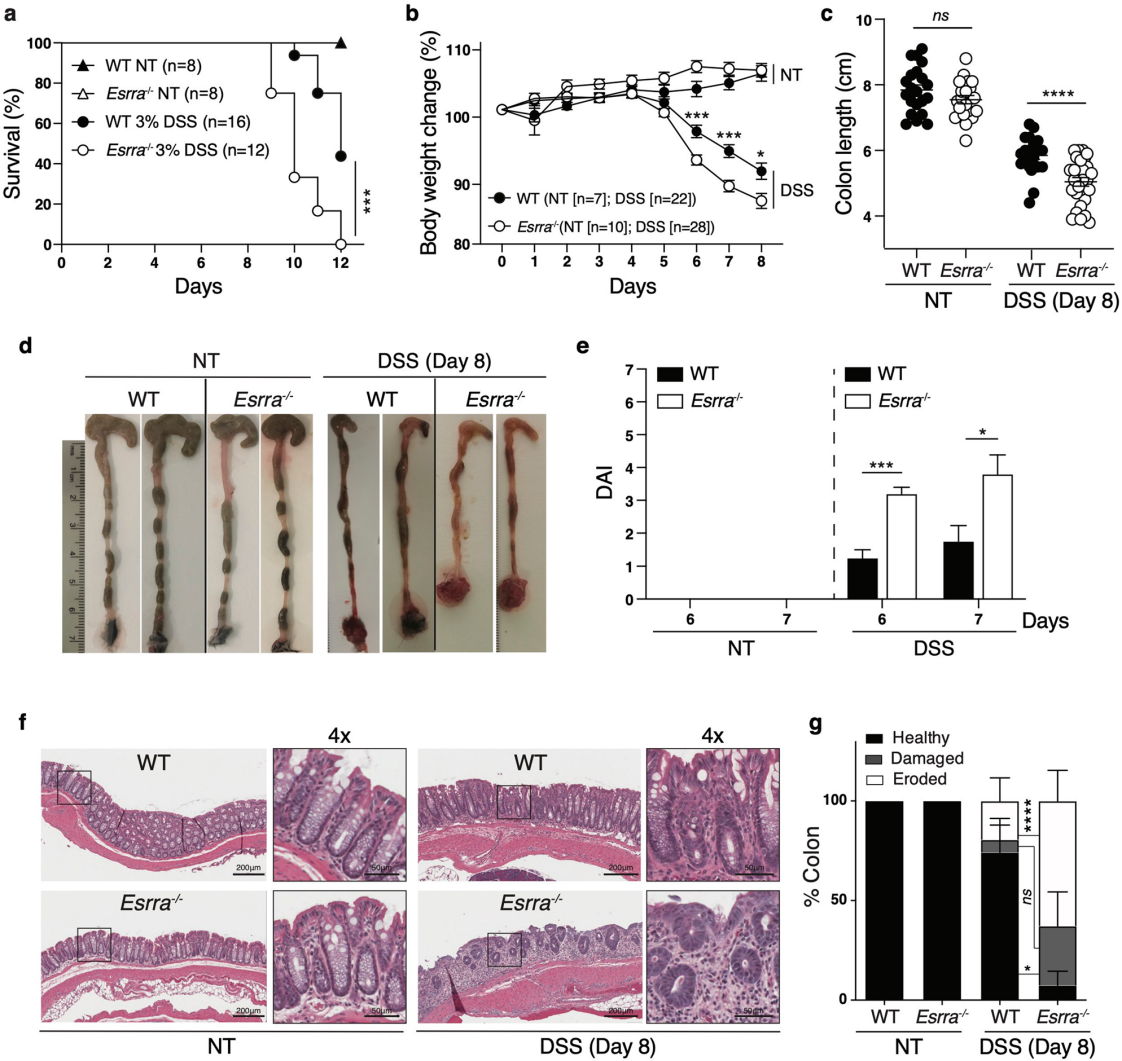
*Esrra*^−/−^ mice exhibited heightened susceptibility to experimental colitis. (**a**) Survival of untreated (NT) WT (n = 8) and *Esrra*^−/−^ (n = 8) mice and WT (n = 16) and *Esrra*^−/−^ (n = 12) mice after 3% DSS treatment. Statistical significance was performed using log-rank (Mantel-Cox) test, ***p < 0.001. (**b**) Body weight loss (%) of untreated (NT) WT (n = 7) and *Esrra*^−/−^ (n = 10) mice and WT (n = 22) and *Esrra*^−/−^ (n = 28) mice after 3% DSS treatment. Statistical significance was performed using Student’s t-test, *p < 0.05, ***p < 0.001. (**c**) Colon lengths of untreated (NT) WT (n = 21) and *Esrra*^−/−^ (n = 21) mice and WT (n = 21) and *Esrra*^−/−^ (n = 28) mice after 3% DSS treatment. Each symbol represents 1 mouse; the horizontal line represents the mean + /- SEM. Statistical significance was performed using Student’s t-test, ****p < 0.0001. *ns,* not significant. (**d**) Representative photographs of colon and cecum from untreated (NT) WT and *Esrra*^−/−^ mice or mice on day 8 after 3% DSS treatment. (**e**) Disease activity index (DAI) of untreated (NT) WT (n = 7) and *Esrra*^−/−^ (n = 10) mice and 3% DSS-treated WT (n = 4) and *Esrra*^−/−^ (n = 5) mice after 6 and 7 days of water or 3% DSS treatment. Statistical significance was performed using Student’s t-test, *p < 0.05, ***p < 0.001. (**f**) Representative Hematoxylin and eosin (H&E) staining of colon sections derived from untreated (NT) WT or *Esrra*^−/−^ mice or mice on day 8 following 3% DSS treatment (magnification ×100, with 4 × zoom). (**g**) Intestinal tissue damage and erosion were quantified by measuring the length of healthy, damaged, eroded sections, and each category expressed as a percentage of colon length. Data represent the mean ± SEM. Statistical analysis was performed using two-way analysis of variance (ANOVA, *p < 0.05, ****p < 0.0001). *ns,* not significant.

To evaluate the role of ERRα in intestinal homeostasis, we first examined its gene expression across 54 tissues obtained from the Genotype-Tissue Expression (GTEx) portal (https://gtexportal.org). This analysis revealed that *ESRRA* expression is highly enriched in normal human colon tissue (Fig. S1a). Furthermore, among the 3 *ESRR* isoforms, *ESRRA* was the predominant isoform expressed in the colon, as assessed in the transverse and sigmoid parts of this tissue (Fig. S1b). To explore the impact of ERR perturbation on colitis in an experimental in vivo model, we subjected wild-type (WT) and *Esrra*^−/−^ mice to 3% DSS in their drinking water for 12 days. Whereas there was no difference in mouse survival in WT or *Esrra*^−/−^ untreated mice, sustained DSS treatment led to significantly heightened mortality in *Esrra*^−/−^ mice compared to WT controls (Fig. 1a). Next, we treated WT or *Esrra*^*-/*^ mice with 3% DSS for 5 days, followed by 3 days of regular drinking water to explore their response to acute DSS-induced injury and follow-up recovery. Treated *Esrra*^−/−^ mice were markedly more susceptible to acute DSS-induced colitis than WT mice, as evidenced by increased body weight loss (Fig. 1b), shortened colon length at endpoint (day 8) (Fig. 1c,d) and more severe disease activity index (DAI) scores, which were based on a combined score of body weight loss, stool consistency, and visual rectal bleeding (Fig. 1e). Histological analysis of colon tissue sections revealed greater tissue injury and loss of colon crypt architecture in treated *Esrra*^−/−^ mice compared to WT mice (Fig. 1f). These phenotypes were not observed in vehicle-treated mice, as *Esrra*^−/−^ mice had similar survival, changes in body weight, colon length and an intact colon histology compared to WT mice (Fig. 1a–f). We next used ImageScope to quantify the extent of colon tissue damage, by measuring the length of healthy, damaged or fully eroded sections and expressing them as a percentage of the full colon length. Damaged crypts were defined as disorganized or incomplete crypts, whereas fully eroded sections were defined by the absence of crypts. Quantitatively, ~ 90% of the colon tissue was damaged or eroded in *Esrra*^−/−^ mice on day 8 post-DSS treatment compared to ~ 30% in WT mice (Fig. 1g). These results suggested that ERRα expression protects against DSS-induced colitis.

**Figure 2:**
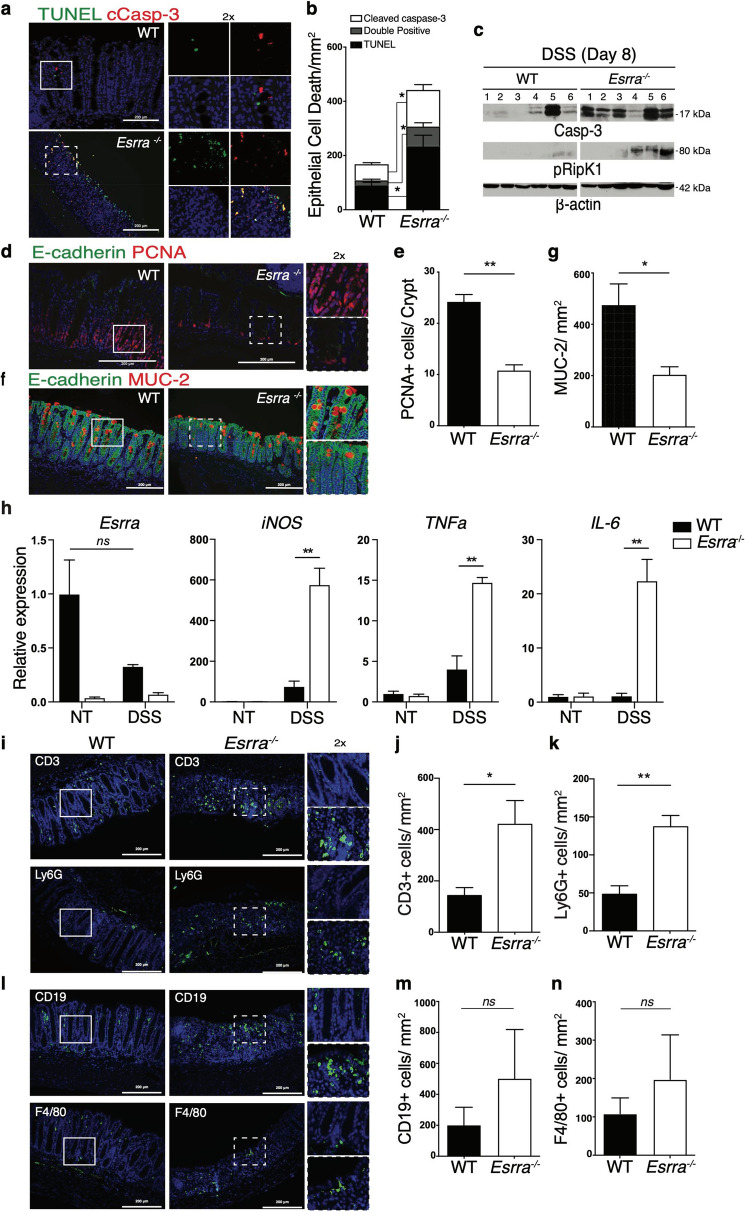
Loss of ERRα results in impaired intestinal epithelial regeneration, increased cell death and exacerbated DSS-induced colonic inflammation. (**a**) Immunofluorescence images of colon sections from WT and *Esrra*^−/−^ mice on day 8 after 3% DSS treatment stained with antibodies against cleaved caspase-3 and TUNEL (terminal deoxynucleotidyl transferase dUTP nick end labeling), and Hoechst to label nuclei. Zoomed images correspond to boxed regions. (**b**) Quantification of cleaved caspase-3 positive, TUNEL positive, and double positive cells, per area (mm^2^) on day 8 following DSS treatment is shown. Data represent the mean ± SEM of 3 mice/genotype. Statistical analysis was performed using Student’s *t*-test, *p < 0.05. (**c**) Western blots depicting cleaved caspase-3, phosphorylated RipK1, and beta-actin levels in colon homogenates from 6 WT and 6 *Esrra*^−/−^ mice on day 8 after 3% DSS treatment. (**d**) Immunofluorescence was performed on colon sections derived from WT and *Esrra*^−/−^ mice on day 8 after 3% DSS treatment stained with antibodies against proliferating cellular nuclear antigen (PCNA) to mark dividing cells, E-cadherin to mark intestinal epithelial cells (IECs), and Hoechst to label nuclei. (**e**) The quantification of PCNA^+^ cells per crypt on day 8 following 3% DSS treatment is shown (5–10 crypts were scored/mouse). Data represent the mean ± SEM of n = 3–4 mice/genotype. Statistical analysis was performed using Student’s *t*-test, **p < 0.001. (**f**) Immunofluorescence images of colon sections from WT and *Esrra*^−/−^ mice on day 8 following 3% DSS treatment stained with antibodies against MUC-2. (**g**) The quantification of MUC-2 positive cells/mm^2^ on day 8 following 3% DSS treatment is shown. Data represent the mean ± SEM of 3 mice/genotype. Statistical analysis was performed using Student’s *t*-test, **p < 0.01. *ns,* not significant. (**i**) Immunofluorescence staining was performed on colon sections derived from WT or *Esrra*^−/−^ mice on day 8 after 3% DSS treatment. The staining was with anti-CD3 antibodies to mark T cells, anti-Ly6G antibodies to mark granulocytes, and Hoechst to label nuclei. (**j**,**k**) Quantification of CD3 + and Ly6G + cells/area (mm^2^) on day 8 following 3% DSS treatment is shown. Data represent the mean ± SEM of n = 3–4 mice/genotype. Statistical analysis was performed using Student’s *t*-test, *p < 0.05, **p < 0.01. (**l**) Immunofluorescence staining was performed on colon sections derived from WT or *Esrra*^−/−^ mice on day 8 after 3% DSS treatment. The staining was with anti-CD19 antibodies to mark B cells, anti-F4/80 antibodies to mark macrophages, and Hoechst to label nuclei. (**m**,**n**) Quantification of CD19+ and F4/80+ cells/area (mm^2^) on day 8 following 3% DSS treatment is shown. Data represent the mean ± SEM of n = 3–4 mice/genotype. Statistical analysis was performed using Student’s *t*-test. *ns,* not significant.

To investigate the underlying mechanisms, we first evaluated the extent of cell death in the colon of DSS-treated animals. Immunofluorescence staining of colon tissue sections with anti-cleaved caspase-3 antibodies and TUNEL showed that loss of ERRα led to increased numbers of early apoptotic (cleaved caspase-3), late apoptotic (double positive) and necrotic (TUNEL) cells (Fig. 2a,b). Consistently, immunoblot analysis of colon homogenates showed increased cleaved caspase-3 and phosphorylated RIPK1, markers of apoptosis and necroptosis, in the colon of *Esrra*^−/−^ mice relative to WT mice (Fig. 2c). Second, we sought to examine the impact of ERRα deletion on colonic epithelial regeneration following tissue damage with DSS. Immunofluorescence staining of colon tissue sections on day 8 post-DSS with anti-PCNA (proliferating cellular nuclear antigen) antibodies revealed markedly reduced IEC compensatory proliferation in the crypts of *Esrra*^−/−^ mice compared to WT mice, suggesting a role of ERRα in tissue restitution (Fig. 2d,e). Last, we evaluated the number of goblet cells, a specialized IEC type responsible for mucus production and depleted in inflammatory contexts such as in IBD patients^31^. Immunofluorescence staining of colon tissue sections on day 8 post-DSS with anti-mucin-2 (MUC-2) antibodies showed a more severe reduction in the number of goblet cells in DSS-treated *Esrra*^−/−^ mice compared to WT mice (Fig. 2f,g). Collectively, our data indicate that ERRα sustains IEC survival and promotes IEC proliferation following injury, and that these homeostatic responses are required for disease tolerance.

### Loss of ERRα results in exacerbated DSS-induced colonic inflammation and alterations in microbiota diversity and composition

To assess the extent of colonic inflammation, we performed qRT-PCR quantification of inflammatory mediators in the colon of untreated or DSS-treated animals, as well as immunofluorescence staining of T cells (anti-CD3), B cells (anti-CD19), macrophages (anti-F4/80) and granulocytes (anti-Ly6G) on colon tissue sections from DSS-treated mice at endpoint. We observed exacerbated expression of inflammatory mediators in colon homogenates of DSS-treated *Esrra*^−/−^ mice compared to WT controls, whereas no differences were observed in untreated mice between the two genotypes (Fig. 2h). We also observed a significant increase in the infiltration of T cells and granulocytes (Fig. 2i–k) and a trend towards increased infiltration of B cells and macrophages (Fig. 2l–n) in the colon of *Esrra*^−/−^ mice compared to WT mice on day 8 post-DSS.

**Figure 3:**
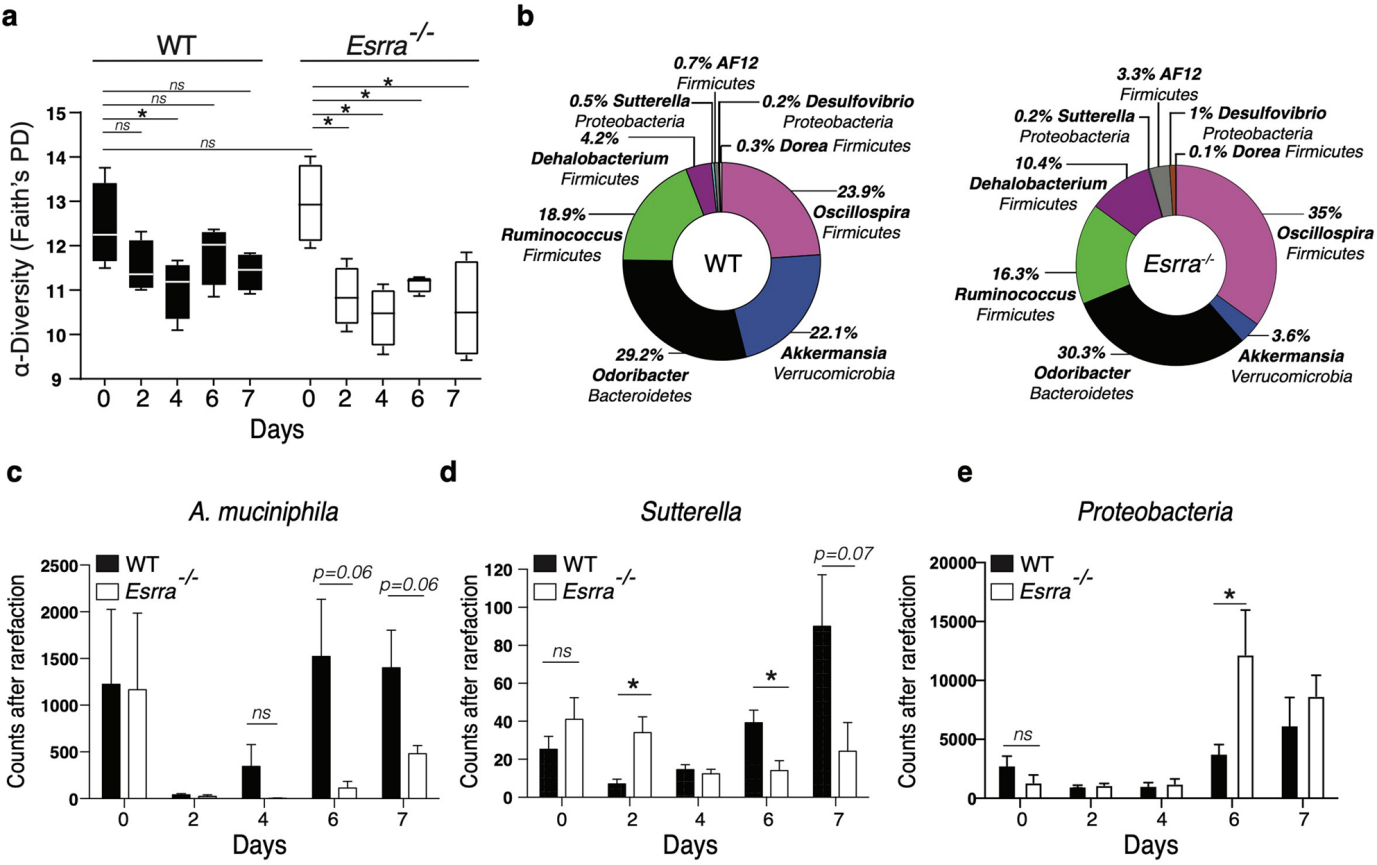
ERRα deficiency leads to alterations in fecal microbiota diversity and composition. (**a**) Analysis of the microbiome alpha diversity in the fecal pellets of WT mice or *Esrra*^−/−^ mice collected at baseline (day 0—untreated) or at different time points after 3% DSS treatment. Kruskal–Wallis for all groups, p < 0.01. (**b**) Bacterial genera (the phylum name is indicated under the genus name) with significant differences in abundance levels (ANCOM test) in the fecal pellets of WT mice versus *Esrra*^−/−^ mice, day 6 is illustrated. Differences in operational taxonomic units (OTUs) between WT and *Esrra*^−/−^ mice at different time points in the DSS experiment in *Akkermansia muciniphila* (**c**), *Sutterella* (**d**), and Proteobacteria (**e**). Data represent the mean ± SEM of n = 4 mice/genotype. Statistical analysis was performed using Student’s *t*-test, *p < 0.05. *ns,* not significant.

We next wished to explore the role of ERRα in the regulation of the host-microbiota crosstalk during the course of colitis. We thus characterized the bacterial microbiota by 16S rRNA sequencing of fecal pellets collected at baseline (day 0) and longitudinally on days 2, 4, 6, and 7 post-DSS in the two genotypes. We elected to examine early time points (e.g. day 2) to determine whether microbiota changes preceded the visual signs of colitis i.e. diarrhea, rectal bleeding, and weight loss (generally observed around day 5 post-DSS). This analysis showed no significant differences in microbiota composition between WT and *Esrra*^−/−^ mice at baseline (day 0). However, a quick decrease in microbial α-diversity was observed as early as day 2 post-DSS treatment in both genotypes, but that was more severe in *Esrra*^−/−^ mice (Fig. 3a). Furthermore, the DSS-induced shift in microbiota composition was markedly influenced by ERRα expression. This was manifested by significant differences in abundance of several genera, according to the ANCOM test, including *Akkermansia*, *Ruminoccocus*, *Oscillospira*, *Odoribacter*, *Desulfovibrio*, *Dehalobacterium* and *Sutterella* between the two mouse genotypes (Fig. 3b). As early as day 2 post-DSS, the levels of *A. muciniphila*, a bacterium associated with a healthy gut, collapsed in both genotypes. However, whereas its levels recovered in WT animals by day 6 post-DSS, they remained depleted in *Esrra*^−/−^ mice (Fig. 3b,c). *Esrra*^−/−^ mice had significantly higher levels of *Sutterella* on day 2 post-DSS, but its levels remained blunted compared to WT mice throughout the course of the treatment (Fig. 3d). As observed in IBD, Proteobacteria expanded in *Esrra*^−/−^ mice as colitis progressed (Fig. 3e). Together, our results indicate that loss of ERRα expression elicits inflammatory responses in the colon and impacts microbial composition prior to the establishment of overt colitis.

### Expression of ERRα in the radio-resistant compartment mediates resistance to DSS-induced colitis

**Figure 4:**
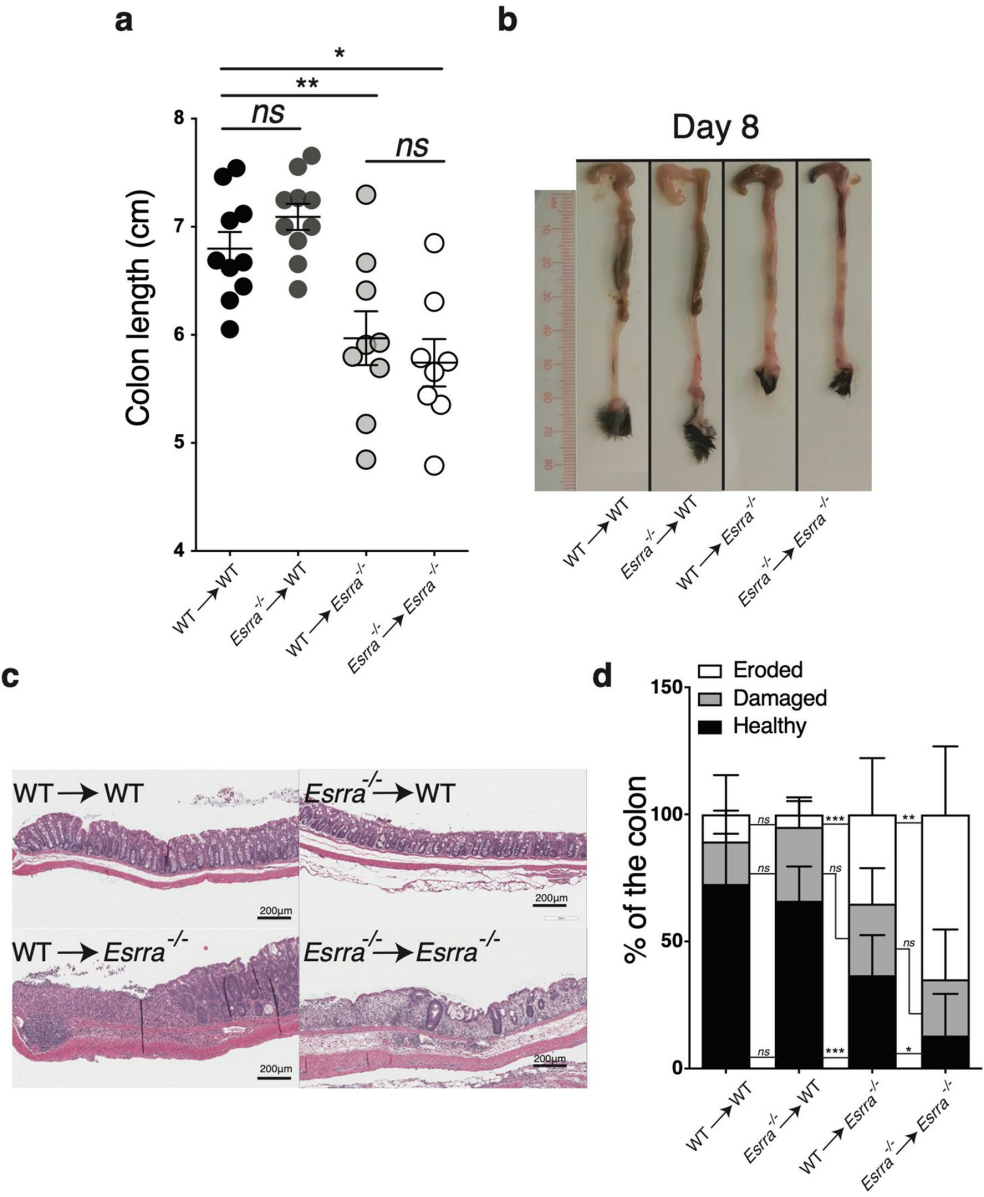
Expression of ERRα in the radio-resistant compartment mediates resistance to DSS colitis. (**a**) Colon length of bone marrow chimera mice on day 8 following DSS treatment. Data represent the mean ± SEM of n = 8–10 mice. Statistical analysis was performed with Student’s *t*-test, *p < 0.05, **p < 0.01. *ns*, not significant. (**b**) Representative photographs of colon and cecum on day 8 following DSS treatment. (**c**) Representative Hematoxylin and eosin (H&E) staining of colon sections derived from bone marrow chimera mice on day 8 following DSS treatment. (**d**) Intestinal tissue damage and erosion of bone marrow chimera mice on day 8 following DSS treatment, quantified by measuring the length of healthy, damaged, eroded sections, and expressed as a percentage of colon length. Data represent the mean ± SEM of 8–10 mice/genotype. Statistical analysis was performed using two-way analysis of variance (ANOVA, *p < 0.05, **p < 0.01, ***p < 0.001. *ns*, not significant).

To determine the cellular compartment in the intestine requiring ERRα expression for countering colitis, bone marrow chimeras were generated by the reconstitution of lethally irradiated mice with bone marrow derived from donor mice of the same or opposite genotype. Twelve weeks after reconstitution, the four chimeric groups were treated with DSS. Our results showed that *Esrra*^−/−^ mice remained highly susceptible to colitis regardless of their bone marrow transplant genotype (WT → *Esrra*^−/−^ or *Esrra*^−/−^ → *Esrra*^−/−^). Of note, the transplant of WT bone marrow did not improve the severely blunted colon length of *Esrra*^−/−^ recipient mice (Fig. 4a,b) but slightly improved colon histology (Fig. 4c,d). Reciprocal transplantation of bone marrow from *Esrra*^−/−^ mice did not transfer the enhanced colitis susceptibility to WT mice (*Esrra*^−/−^→WT), which were equivalent to WT controls (WT→WT), as observed at the level of colon length (Fig. 4a,b) and histopathology (Fig. 4c,d). These findings indicate that ERRα acted primarily in the radio-resistant compartment of the colon to mediate its protective effects against DSS-induced colitis.

### The ERRα-dependent colon and IEC transcriptomes in colitis

**Figure 5:**
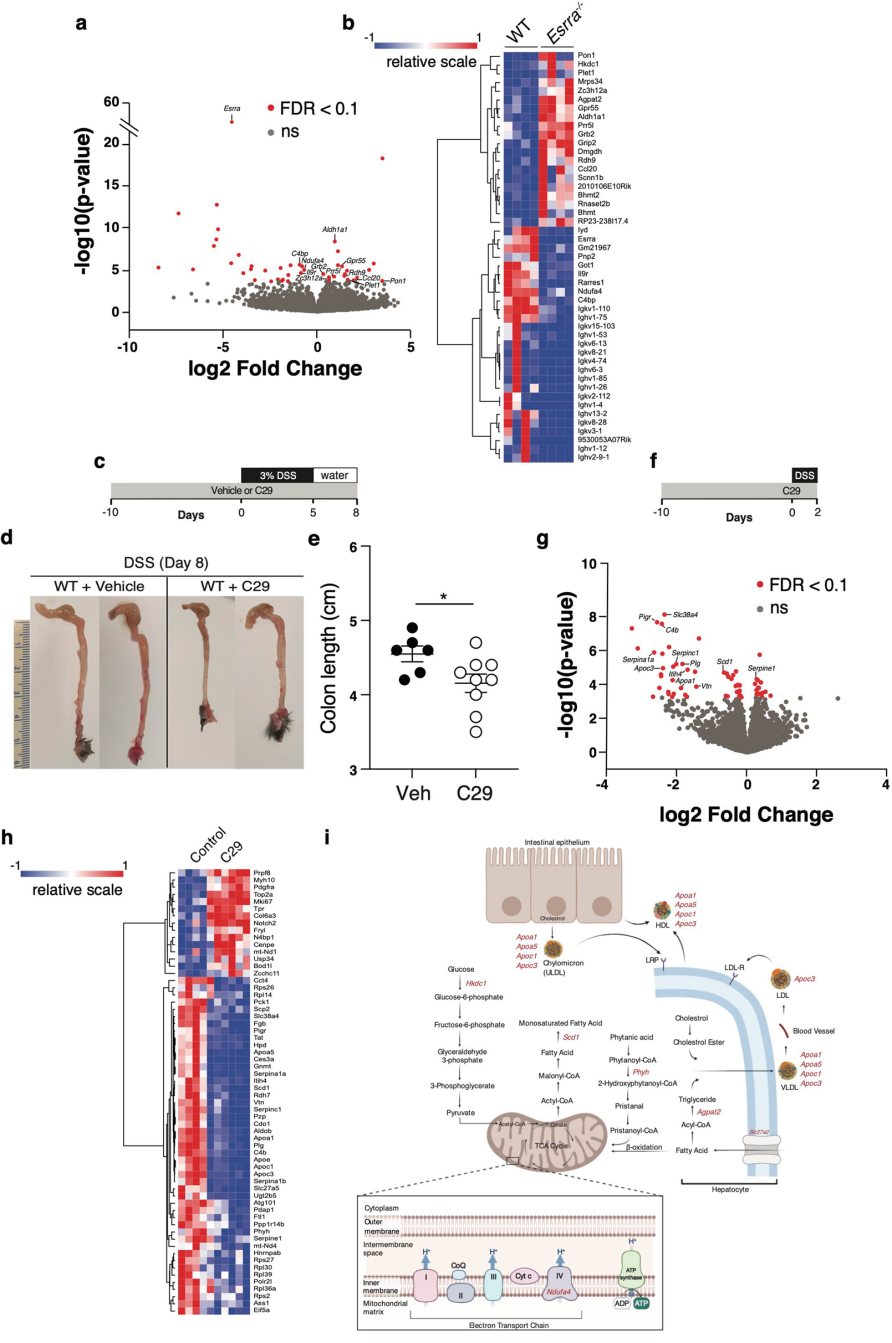
The ERRα-dependent colon and IEC transcriptomes in colitis. (**a**) Volcano plot of RNA sequencing data obtained from DEseq2 analysis of the colon transcriptome of WT or *Esrra*^−/−^ mice on day 8 post-DSS. Genes that are significantly differentially expressed (FDR < 0.1) are highlighted in red. Data represent 4 mice/genotype. Volcano plots were generated using DEseq2 (v.1.28.1, https://bioconductor.org/) in R (version 3.5, https://cran.r-project.org/) with packages ggplot2 (v.3.3.3). (**b**) Heatmap of upregulated (red) or downregulated (blue) genes in the colon of WT or *Esrra*^−/−^ mice on day 8 following DSS treatment. Heatmaps were generated using the online version of Morpheus from the Broad Institute (https://software.broadinstitute.org/morpheus/). (**c**) Schematic representation of the experimental procedure of DSS-induced colitis following acute inhibition of ERRα. WT mice were either pre-treated with the ERRα inverse agonist C29 or vehicle control for 10 days then subjected to 3% DSS in their drinking water for 5 days, followed by regular drinking water for an additional 3 days (days 5–8). (**d**) Representative photographs of colon and cecum from vehicle- or C29-treated WT mice on day 8 following 3% DSS treatment. (**e**) Colon length of vehicle (n = 6) or C29 (n = 9) treated WT mice after 3% DSS treatment. Statistical significance was performed using Student’s *t-*test, *p < 0.05. (**f**) Schematic representation of the experimental procedure of DSS-colitis following acute inhibition of ERRα. WT mice were either pre-treated with the ERRα inverse agonist C29 or vehicle control for 10 days then subjected to 3% DSS in their drinking water for 2 days. (**g**) Volcano plot of RNA sequencing data obtained from DEseq2 analysis of the IEC transcriptome of WT mice treated with C29 (n = 6) or vehicle control (n = 4). Significantly differentially expressed (FDR < 0.1) genes are depicted in red. Volcano plots were generated using DEseq2 (v.1.28.1, https://bioconductor.org/) in R (version 3.5, https://cran.r-project.org/) with packages ggplot2 (v.3.3.3). (**h**) Heatmap of upregulated (red) or downregulated (blue) genes in IECs isolated from mice injected with compound C29 (n = 6) or vehicle control (n = 4). Heatmaps were generated using the online version of Morpheus from the Broad Institute (https://software.broadinstitute.org/morpheus/). (**i**) Schematic illustration of metabolic genes downregulated by perturbation of ERRα (red) following DSS treatment.

To explore the ERRα transcriptional program in colitis, we first performed bulk RNA sequencing (RNAseq) of the colon from WT or *Esrra*^−/−^ mice on day 8 post-DSS. The genes regulated by ERRα at endpoint are depicted in a volcano plot (Fig. 5a) and a heatmap (Fig. 5b). Other than *Esrra* (gene for ERRα), a total of 46 genes, implicated in diverse biological processes including immunity, metabolism, and wound healing, were identified to be differentially expressed (DE) between the two genotypes (FDR < 0.1). These included direct ERRα transcriptional targets (e.g. *Rhdh9*, *Aldh1al*, *Rarres*, *Agpat2, Got1, Ndufa4*, *Pon1*, *Gpr55*, *Prr5l*, *Grb2, Grip2, Scnn1b*, *Bhmt, Bhmt2* and *Il9r*)^32, 33^*.* Several genes of the humoral immune response were downregulated in *Esrra*^−/−^ mice, including immunoglobulin genes and *C4bp*, a regulator of the complement cascade. In contrast, expression of the chemokine Ccl20 and regnase-1 (*Zc3h12a*), a negative regulator of TLR and IL-1R mediated cytokine production^34^, were upregulated in *Esrra*^−/−^ mice. Several metabolic genes were also DE between the two genotypes. These included *ndufa4* (oxidative metabolism), *agpat2* (lipid metabolism), *got1* (amino acid and urea metabolism)*, bhmt* and *bhmt2 (*homocysteine metabolism), *hkdc1* (glucose metabolism), *lyd* (hormone metabolism), *dmgdh* (choline metabolism) and *rdh9* and *ald1a1* (retinoic acid metabolism). The remaining DE genes included *plet1*, involved in wound healing, and *pon1* encoding Paraoxonase 1, an antioxidant downregulated in IBD^35^.

Based on ERRα’s protective role in the radio-resistant compartment of the intestine, the abundance of IECs compared to other radio-resistant cells, and the key role of the ERRα co-activator PGC1α in IECs^12^, we next decided to explore the ERRα-dependent IEC transcriptome. Further to avoid adaptation mechanisms arising in *Esrra*^−/−^ mice, and as a clinically relevant intervention, we opted to study the effects of acute inhibition of ERRα with an inverse agonist, named Compound 29 (C29)^36^ in WT mice. As observed in *Esrra*^−/−^ mice, pre-treatment of WT mice with C29 led to enhanced colitis severity on day 8-post DSS administration compared to vehicle-treated mice (Fig. 5c–e). To maximize the discovery of genes involved in intestinal homeostasis or disease induction, we performed the IEC RNAseq analysis at an earlier time point following DSS treatment (day 2). A total of 62 DE genes were found between C29 and vehicle-treated mice (FDR < 0.1) (Fig. 5f–h), including direct ERRα transcriptional targets. Among these were genes that play a role in lipid metabolism (Fig. 5i), many of which have been previously implicated in IBD^37, 38^. These included *Apoc3, Apoa1, Scd1,* components of lipoproteins (*Apoc1* and *Apoa5)*, *Slc27a5*, involved in fatty acid elongation and synthesis, and *Phyh*, responsible for alpha-oxidation of peroxisomes. These lipid metabolism genes were found downregulated following acute inhibition of ERRα. C29 also impacted genes involved in immunity, blood coagulation, amino acid metabolism, protein transport, cell adhesion and spreading, and RNA synthesis. Immune-related genes downregulated by ERRα inhibition included *Pigr*, *C4b*, and *Itih4*. The remaining downregulated genes included the organic solute carrier *Slc38a4* known to transport amino acids, *Serpinc1*, *Serpine1,* and *Plg*, known regulators of the blood coagulation cascade, and *Vtn*, involved in mucosal wound healing. Collectively, our data reveal a critical role of ERRα in intestinal homeostasis through a transcriptional program regulating several metabolic and immunity-related genes, with demonstrated links to IBD as discussed below.

## Discussion

ERRα controls a wide transcriptional network pertaining to cellular energy metabolism, mitochondrial function, autophagy and wound healing, among others^32, 33, 39^. As dysfunctional mitochondria release danger-associated molecular patterns (DAMPs), including ROS, oxidized mtDNA and cardiolipin, it is plausible that the accumulation of these DAMPs in ERRα-deficient mice promotes IEC death and inflammatory tissue damage. Indeed, ROS production is increased in experimental colitis and correlates with disease severity^12^. ERRα has been reported to induce anti-oxidant protective genes^40^, and decreased antioxidant capacity has been described in IBD patients^41^. We have observed a decrease in the expression of *pon1*, a gene linked to IBD that normally mediates both antioxidant and anti-inflammatory activities in IECs^42^. The severe colitis phenotype associated with ERRα loss may also be due to a failure to provide adequate energy supply to sustain colonocyte survival and barrier integrity, as maintenance of tight junctions (TJ) between IECs is energy dependent^43^. Lipid metabolism, in particular *apoa1* gene activity, supports TJ recovery after damage^44^. ERRα may also be exerting its protective role through autophagy, which is central for intestinal homeostasis^7, 45, 46^.

Although the exacerbated inflammation in *Esrra*^−/−^ mice could be secondary to their marked epithelial tissue damage, the inflammatory infiltrates and mediators found upregulated in *Esrra*^−/−^ mice are implicated in IBD pathogenesis. In particular, IL-6 levels are associated with disease activity in IBD patients and the risk of relapse^47, 48^. Similarly, iNOS overexpression and increased NO production are linked to IBD disease severity^49^. Our results show that ERRα-deficiency resulted in exacerbated iNOS induction in the colon of colitic mice. NO impairs beta-oxidation in IECs leading to oxygen accumulation in the intestinal lumen and microbiota dysbiosis, including the expansion of pathogenic *Enterobacteriaceae* of the Proteobacteria phylum^50^. It is plausible that ERRα limits the bioavailability of oxygen in the lumen of the colon by promoting FAO. This hypothesis is consistent with our results showing a downregulation of lipid metabolism genes in the colonocytes of *Esrra*^−/−^ mice and an expansion of Proteobacteria. Furthermore, we observed a dramatic depletion of *Akkermansia* in *Esrra*^−/−^ mice upon induction of colitis. *Akkermansia* utilises mucin as an energy source and releases metabolic by-products including SCFA, e.g. butyrate, among other factors for neighbouring gut symbionts^51^. Butyrate is catabolized by colonic epithelial cells and provides the bulk of intestinal energy demand, thus, is important in the maintenance of colonocyte survival and mucosal health^52^. Indeed, animals exhibited severe signs of experimental colitis when butyrate oxidation was inhibited^53^. Accordingly, a decrease in *Akkermansia* read counts in *Esrra*^−/−^ mice might contribute to the disruption of intestinal homeostasis by limiting butyrate production and availability.

The colon transcriptome of mice, at colitis endpoint, might not reflect direct functions of ERRα in intestinal homeostasis, as it can be affected by the inflammatory environment. Nonetheless, such analysis confirmed the heightened inflammatory pathology observed in *Esrra*^−/−^ mice. Several IBD-implicated genes were identified to be DE in *Esrra*^−/−^ mice. For instance, *Ccl20*, which encodes the sole ligand of CCR6, was upregulated in *Esrra*^−/−^ mice. This chemokine is involved in lymphocyte trafficking and chronic intestinal inflammation^54^. Consistently, *Ccr6*^−/−^ mice have been reported to be less susceptible to DSS colitis^55^. Our results show that *Ndufa4* that encodes a subunit of complex I of the respiratory chain was downregulated in DSS-treated *Esrra*^−/−^ mice. It has been previously demonstrated that Ndufa4 dysregulation leads to impairment of mitochondrial respiration and the generation of ROS^56^, which further fuels the inflammatory vicious cycle.

By inhibiting ERRα acutely with the inverse agonist C29^36^, we aimed to abolish ERRα activity at an early time point in the DSS treatment regimen to explore causal mechanisms. This analysis highlighted an important role of ERRα-transcriptional targets involved in lipid energy metabolism (Fig. 5i). *Apoc3* underwent the largest decrease in gene expression by C29. It has been previously shown that IEC-specific deletion of *Apoc3* resulted in increased susceptibility to acute colitis^57^, and expression of this gene is significantly reduced in IBD patients^37^. More recently, it was demonstrated that ApoC-III-containing lipoproteins regulate intestinal immune tolerance through Tregs and IL-10^58^. Downregulation of *Apoc3* in C29-treated mice might thus lead to impaired immune tolerance. The *apoa1* gene is similarly downregulated by C29 in our experiments. ApoA-I, the main component of high-density lipoprotein (HDL), is anti-inflammatory. In enterocytes, HDL and apoA-I supress NF-κB-dependent induction of proinflammatory cytokines, and apoA-I null mice were previously reported to be susceptible to colitis^59^. ApoA-I regulates lipid transport in the intestine^60^ and is required to maintain IEC TJ^44^. The *Scd1* gene, which encodes an enzyme involved in the synthesis of oleic acid, was also downregulated following acute inhibition of ERRα in our experiments. Consistently, previous studies have shown that *Scd1* deletion from the intestinal epithelium led to increased gut inflammation, a phenotype that could be rescued by dietary supplementation of oleic acid^61^.

**Figure 6:**
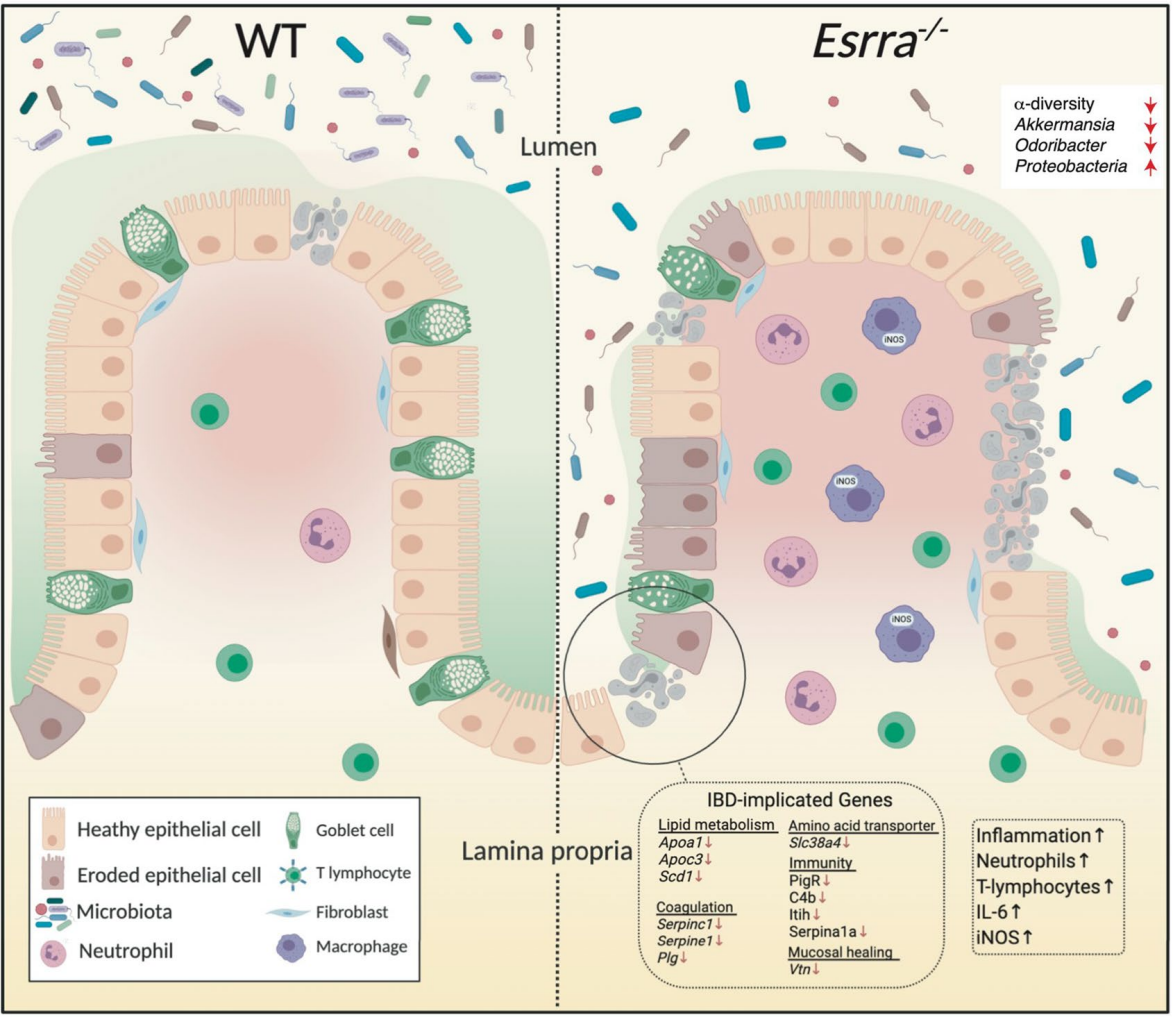
Model illustrating the impact of ERRα-deficiency in experimental colitis. (**a**) The colon of a WT or an ERRα-deficient condition in experimental colitis. ERRα-deficiency results in a reduction in microbiota α-diversity, a collapse of bacteria associated with a healthy gut such as *Akkermansia* and an increase in Proteobacteria. This is accompanied by heightened epithelial tissue damage and erosion and an exacerbated intestinal inflammation characterized by increased immune infiltrates (neutrophils, T-lymphocytes) and inflammatory mediators (IL-6 and iNOS). IBD-implicated genes downregulated by ERRα inhibition are shown.

Additional C29-modulated genes included genes involved in immunity, blood coagulation, amino acid metabolism, protein transport, cell adhesion and spreading, and RNA synthesis. A reduction of Polymeric immunoglobulin receptor (*Pigr*) gene expression was observed following acute inhibition of ERRα. Interestingly, *Pigr* downregulation is a biomarker of CD severity^62^. We show that *Slc38a4*, which encodes an organic solute carrier known to transport amino acids, was also downregulated by C29, consistent with previous findings in IBD patients^63^. The *C4b* gene*,* involved in the classical complement activation pathway, has a critical role in maintaining tissue homeostasis through microbial elimination. *C4b* genes were previously linked to pediatric IBD and their deficiency was shown to increase susceptibility to infections^64^. *Serpinc1*, *Serpine1,* and *Plg*, regulators of the blood coagulation cascade and necessary for mucosal healing^65^, were downregulated by C29. *Vtn* has also been implicated in mucosal healing in the recovery phase of DSS-colitis^66^. *Serpina1a*, which encodes alpha-1 antitrypsin, is known to exert an anti-inflammatory role by neutralizing neutrophil elastase. Previous studies have demonstrated elevated elastase activity in patients with IBD and experimental models of colitis^67, 68^. This is consistent with increased neutrophilic infiltrates shown in our data. Overall, the biological processes regulated by ERRα may act in synergy to protect the intestine and counter colitis (Fig. 6).

Consistent with our findings in this manuscript showing that genetic disruption of ERRα enhances colitis pathology in a murine model, *ESRRA* encoding human ERRα was recently found to be downregulated in IBD by the PROTECT study, which examined 428 treatment naïve UC patients and identified a core rectal UC gene expression signature^69^. Among the DE genes, *ESRRA* was significantly downregulated in UC compared to non-IBD controls (*p* = 1.10E−09), and gene set enrichment analysis (GSEA) of the altered genes in UC revealed a significant enrichment of genes regulated by ERRα, specifically in IECs^69^. Importantly, this study pointed to mitochondriopathy and decreased aerobic tricarboxylic acid (TCA) cycle and metabolic functions (processes regulated by ERRα) as key features of UC. In contrast to IBD, colonic ERRα expression is upregulated in colorectal cancer (CRC) and is associated with poor disease prognosis^70, 71^. ERRα may thus be considered as a double-edge sword; it protects the intestine by promoting colonocytes survival and inducing compensatory proliferation after injury. On the other hand, its overexpression might exacerbate these processes leading to CRC.

In summary, this study reports a critical role of ERRα in the intestinal epithelium, validating it as a potential therapeutic target for clinical intervention in IBD.

## Materials and methods

### Animal strains

WT and ERRα-deficient (*Esrra*^−/−^) mice on a C57B1/6J background were bred and maintained at the McGill Comparative Medicine and Resources Centre. *Esrra*^−/−^ mice were generated as previously described and were maintained by crossbreeding^72^. All animals used were 8–12 weeks old male mice housed under pathogen-free conditions and were fed standard laboratory chow. The WT and *Esrra*^−/−^ mouse colonies were generated from littermate parents. For most DSS experiments, including the bone marrow chimera experiments, mice of the two genotypes were co-housed for 2 weeks prior to DSS treatment to normalize potential differences in their microbiota. All experiments were approved by, and performed under guidelines, of the animal ethics committee of McGill University (Canada). This study is reported in accordance with ARRIVE guidelines.

### Experimental colitis

Acute experimental colitis was induced by administering 3% (w/v) DSS (36,000–50,000 kDa, MP Biomedicals Cat#160110) in the drinking water for 5 days (the DSS solution was renewed every second day) and subsequently replaced with regular water for an additional 3 days to allow for recovery. For the survival experiments, 3% (w/v) DSS was administered continuously for 12 days. Disease activity index (DAI) scores were based on a combined score for body weight loss, stool consistency, and visual rectal bleeding ranging from 0–7. For body weight loss, a score of 0–4 was given: 0, 0–5%; 1, 5–10%; 2, 10–15%; 3, 15–20%; and 4, > 20%. For stool consistency, a score of 0–3 was given: 0, normal; 1, mild diarrhea; and 2, severe diarrhea. A score of 0 or 1 was given for the visual absence or presence, respectively, of rectal bleeding. For all experiments, we processed the colons following a defined dissection protocol applied to all mice, as follows: the 1st cm from the anal verge is discard, the 2nd cm for RNA extraction, the 3rd for protein extraction, the 4th for organ culture, and the remaining tissue (~ 2–3 cm) is used for histology.

### Bone marrow chimera

C57BL/6 and *Esrra*^−/−^ mice were lethally irradiated with 450 rads twice in a 3-h interval using an X-ray RS-2000 Biological irradiator. Lethally irradiated mice were i.v. injected with 10^7^ bone marrow cells after red blood cell depletion from indicated donors. Mice received 50 mg/mL enrofloxacin (Baytril) in their drinking water 3 days before the irradiation and for 3 weeks post-irradiation. 8 weeks post bone marrow transplant, engraftment was verified by FACS staining and was confirmed as > 90% in recipient mice.

### Acute inhibition of ERRα

C57BL/6 WT mice were i.p. injected daily for 10 days with vehicle or 10 mg/kg of the ERRα inverse agonist compound 29 (C29)^36^ (Omegachem Inc.) in Ringer’s solution (containing 5.2% polyethylene glycol and 5.2% tween 80) prior to start of the DSS experiments and continued until the end of the experiments.

### Isolation of intestinal epithelial cells

To isolate the IECs, colons were washed with cold PBS (Wisent, Cat#311-425-CL), cut into small pieces in RPMI (Wisent, Cat.350-000-CL) containing 5 mM EDTA (Fisher, Cat.BP2482-500), 3% FBS (Wisent, Cat# 350-000-CL), and DTT (Fisher, Cat.BP172-5) and placed in a shaking (170 RPM) incubator for 45 min at 37 °C. Following filtration in 100 μM strainers (Fisher, Cat.#10282631), the supernatant was centrifuged at 4 °C at 1500 rpm, and cells were resuspended in 30% (vol/vol) Percoll (Sigma, Cat#P4937-100ML). The cells were spun again at 4 °C and the top layer isolated and resuspended in fresh RPMI (Wisent, Cat.350-000-CL). Centrifugation was repeated and RNA was extracted from pelleted cells using miRNeasy kit (Qiagen, Cat. # 217004) with RNase-free DNase Set (Qiagen, Cat. #79254).

### Hematoxylin and Eosin (H&E) staining and immunofluorescence

Pieces of 1 cm^2^ of colon tissue were paraffin embedded following an overnight fixation in 10% buffered formalin. Sections (4 μm thick) were cut onto glass slides and stained with H&E. Images were captured using a ScanScope XT digital scanner and analyzed with the ImageScope software. Intestinal tissue damage and erosion were quantified by dividing the length of healthy, damaged or fully eroded sections by that of the full colon. For immunofluorescence, tissue sections were rehydrated by sequential incubation in ethanol solutions of different concentrations (100%, 90%, 70%, 50%; 5 min each). For antigen retrieval, the slides were incubated in 0.1 M citrate buffer (pH 6.0) at 95 °C for 15 min. For permeabilization, the slides were next incubated in 0.25% Triton X-100 for 20 min at room temperature. For TUNEL staining, Click-iT TUNEL Alexa Fluor 647 (Invitrogen, Cat# C10247) was used according to the manufacturer’s instructions. Prior to incubation with primary antibodies, the slides were incubated in a solution of 10% FBS, 3% BSA blocking solution for 30 min at 37 °C. Primary antibodies diluted in a PBS solution with 3% BSA were used for overnight incubation of slides at room temperature. The following antibodies were used: PCNA (Abcam, Cat# AB2426; RRID:AB_303062), cleaved caspase-3 (R&D Cat# AF835; RRID:AB_2243952), E-cadherin (BD Bioscience Cat# 610182; RRID:AB_397581), F4/80 (Abcam, Cat #ab6640; RRID:AB_1140040), Gr-1 (BD, Cat #557661; RRID:AB_396775), CD19 (eBioscience, Cat# 12-0191-81; RRID:AB_465577), CD3 (eBioscience, Cat# 45-0031-82; RRID:AB_906226), Ly6G (Abcam, Cat #ab210204) and Muc2 (Santa Cruz Biotechnology, Cat#sc-15334; RRID:AB_2146667). The slides were then washed with PBS and incubated with secondary antibodies for 1 h at room temperature. The secondary antibodies were conjugated with Alexa Fluor 488, 594 or 647 (Molecular Probe). After washing with PBS, Hoechst 33342 (Invitrogen, Cat# H3570) staining was added. A Zeiss Axioscope microscope with its AxioVision software (version 4.9.1) and a high-resolution AxioCam (Carl Zeiss Microscopy) were used for image acquisition at the McGill University Life Sciences Complex Advanced BioImaging Facility (ABIF). Image processing was performed using ImageJ 1.46 (National Institute of Health) on epifluorescence images after background clearance using the ‘BG subtraction from ROI’ plugin. Staining-positive cells were counted manually and quantified by crypt area (mm^2^).

### Western blot

1 cm^2^ of colon tissue was dissected, washed in PBS and homogenized in complete buffer B150 (20 mM Tris–HCl pH 8.0, 150 mM KCl, 10% glycerol, 5 mM MgCl2, and 0.1% NP40) containing protease and phosphatase inhibitors (Roche Applied Science, Cat# 11836153001 and Sigma Cat# S7920, 71768, G6376). Proteins were migrated on an SDS-PAGE gel and transferred to a nitrocellulose membrane. Blots were incubated with primary antibodies against cleaved caspase-3 (Cell Signaling Cat# 9661; RRID:AB_2341188), anti-RIPK1 phospho-S166 (Cell Signaling Cat# 31122; RRID:AB_2799000), and β-actin (Sigma, Cat# A1978; RRID:AB_476744), followed by corresponding species secondary antibodies. Uncropped images of the western blots are shown in Supplementary Fig. 2.

### RNA sequencing and analysis

1 cm^2^ of colon tissue was dissected and washed in PBS. mRNA was extracted from total colon or purified IEC with the miRNeasy kit (Qiagen Cat. # 217004). Assessment of RNA quality and quantity was performed using Agilent Bioanalyzer 2100. 1 μg RNA was used for RNA sequencing at Novogene Corporation Inc, Beijing, China. Between 50 and 66 million raw reads were generated from each library. Bioconductor DEseq2 package (v.1.24) was used to calculate differential gene expression. Fragments per kilobase million (FPKM) were log transformed and normalized. R packages ggplot2 was used for the generation of the volcano plots. Heatmaps were generated using the online version of Morpheus from the Broad Institute (https://software.broadinstitute.org/morpheus/). Benjamini and Hochberg correction for multiple comparisons (at least one sample’s FPKM ≥ 1) was used for statistical analysis with an FDR-adjusted p-value of 0.1 for the heatmaps or 0.05 for the volcano plots.

### Microbiota DNA sequencing and analysis

Fecal DNA was collected in 1.5 ml eppendorf tubes (Fisherbrand) and stored at − 80 °C until preparation. Total DNA was extracted using QIAmp PowerFecal DNA Kit (Cat#:12830-50) according to the manufacturer’s instructions (QIAmp DNA Stool Handbook 08/2017). DNA concentrations were measured with a NanoDrop Spectrometer and stored at − 80 °C. Samples (48 total) of gDNA (10 μg/μl) were sequenced at Genome Quebec, according to their protocol provided here. DNA was amplified using primer pair 341F (CCTACGGGNGGCWGCAG) and 805R (GACTACHVGGGTATCTAATCC) from Illumina’s 16S library with sample-specific barcodes. These amplify the 16S V4 variable region of the rRNA gene. For the 48 samples, a total of 5,153,394 reads, 2,576,697,000 bases, with an average quality of 36 was obtained. The 464 bp PCR products were sequenced on MiSeq PE250 Illumina platform (Illumina Inc., San Diego, CA, USA) generating two FASTQ files per sample. Paired reads from MiSeq were trimmed of primers, filtered for high quality (phred score > 30) and merged. Reads with at least 1 “N” were discarded, and sequence chimeras were removed using Uchime2 against the "Gold" database^73^. Taxonomic affiliation of 16S sequencing data was performed using the QIIME2 pipeline. Quality filtered sequences from pre-processing were denoised and underwent further QC filtering by DADA2 to create representative sequences for each amplicon sequence variant (AVS), and a feature table which indicates quantity of reads of each AVS observed in each sample. Feature classifier was used to assign likely taxonomies to reads through a model pretrained by a Naive Bayes classifier on GreenGenes database with 99% OTUs. Data were rarefied using the samples with the smallest number of reads and then subjected to alpha-diversity analysis employing Faith’s phylogenetic diversity. Kruskal–Wallis was calculated between all groups together and for pairwise comparisons. To find significant differences at the genera taxonomy level, ANCOM volcano plots were drawn to identify features that are differentially abundant across sample groups^74^.

### Quantitative real-time PCR

For qRT-PCR, 1 cm^2^ of mouse colon tissue was dissected and washed in PBS. Total RNA was isolated using the RNeasy Mini Kit (Qiagen). cDNA was made from 1 μg of RNA by reverse transcription with Random Primer Mix, dNTPs, 5X ProtoScript II RT Reaction buffer, DTT, RNAse inhibitor and ProtoScript II Reverse Transcriptase (NEB). cDNA was amplified by qRT-PCR using SYBR Green Master Mix (Roche) and a LightCycler 480 instrument (Roche) with specific primers. Relative quantification was normalized to *RPLP0* expression using the 2^−ΔΔCT^ method. Specific primers used were:

- *Esrra*:
- forward 5′-CCAGAGGTGGACCCTTTGCCTTTC-3′
- reverse 5′-CACCAGCAGATGCGACACCAGAG-3′
- *iNOS*:
- forward 5′-GTGAAGAAAACCCCTTGTGCTG-3′
- reverse 5′-CTCACATACTGTGGACGGGTCG-3′
- *TNFa*:
- forward 5′-CTTCCAGAACTCCAGGCGGTGC-3′
- reverse 5′- CATAGAACTGATGAGAGGGAGGC-3′
- *IL-6*:
- forward 5′-AAGTGCATCATCGTTGTTCA-3′
- reverse 5′-GAGGATACCACTCCCAACAG-3′
- *RPLP0*:
- forward 5′-GCAGCAGATCCGCATGTCGCTCCG-3′
- reverse 5′-GAGCTGGCACAGTGACCTCACACGG-3′.

### Statistical analysis

Data is represented as mean ± standard error of the mean. Two-tailed Student's t test and ANOVA were used for evaluating statistical significance between groups. *P < 0.05; **P < 0.01; ***P < 0.001; ****P < 0.0001; N.S., not significant. The ANCOM test was used to identify microbiota features that are differentially abundant between genotypes.

## Supplementary Information

Supplementary Information 1.

Supplementary Information 2.
