# Supplementary material for: Estrogen-related receptor alpha (ERRα) is a key regulator of intestinal homeostasis and protects against colitis
Source: Sci Rep. 2021 Jul 23;11:15073. doi: 10.1038/s41598-021-94499-5 (PMC8302669; doi:10.1038/s41598-021-94499-5)
Supplement: Supplementary file 1 — Supplementary Information 1. [file 41598_2021_94499_MOESM1_ESM.docx]

**Supplemental Figure 1.** *ESRRA* expression is highly enriched in normal human colon tissue. **(a)** Violin plots of human RNA-seq gene expression profiles of *ESRRA* across 54 tissues obtained from the Genotype-Tissue Expression (GTEx) portal (<https://gtexportal.org>). **(b)** Relative median expression values of ERR-encoding isoforms in human colon tissues from the GTEx portal.

**Supplemental Figure 2.** Uncropped images of the western blots shown in Fig. 2c.
