# Supplementary material for: Estrogen-related receptor alpha (ERRα) is a key regulator of intestinal homeostasis and protects against colitis
Source: Sci Rep. 2021 Jul 23;11:15073. doi: 10.1038/s41598-021-94499-5 (PMC8302669; doi:10.1038/s41598-021-94499-5)
Supplement: Supplementary file 2 — Supplementary Information 2. [file 41598_2021_94499_MOESM2_ESM.pdf]

a

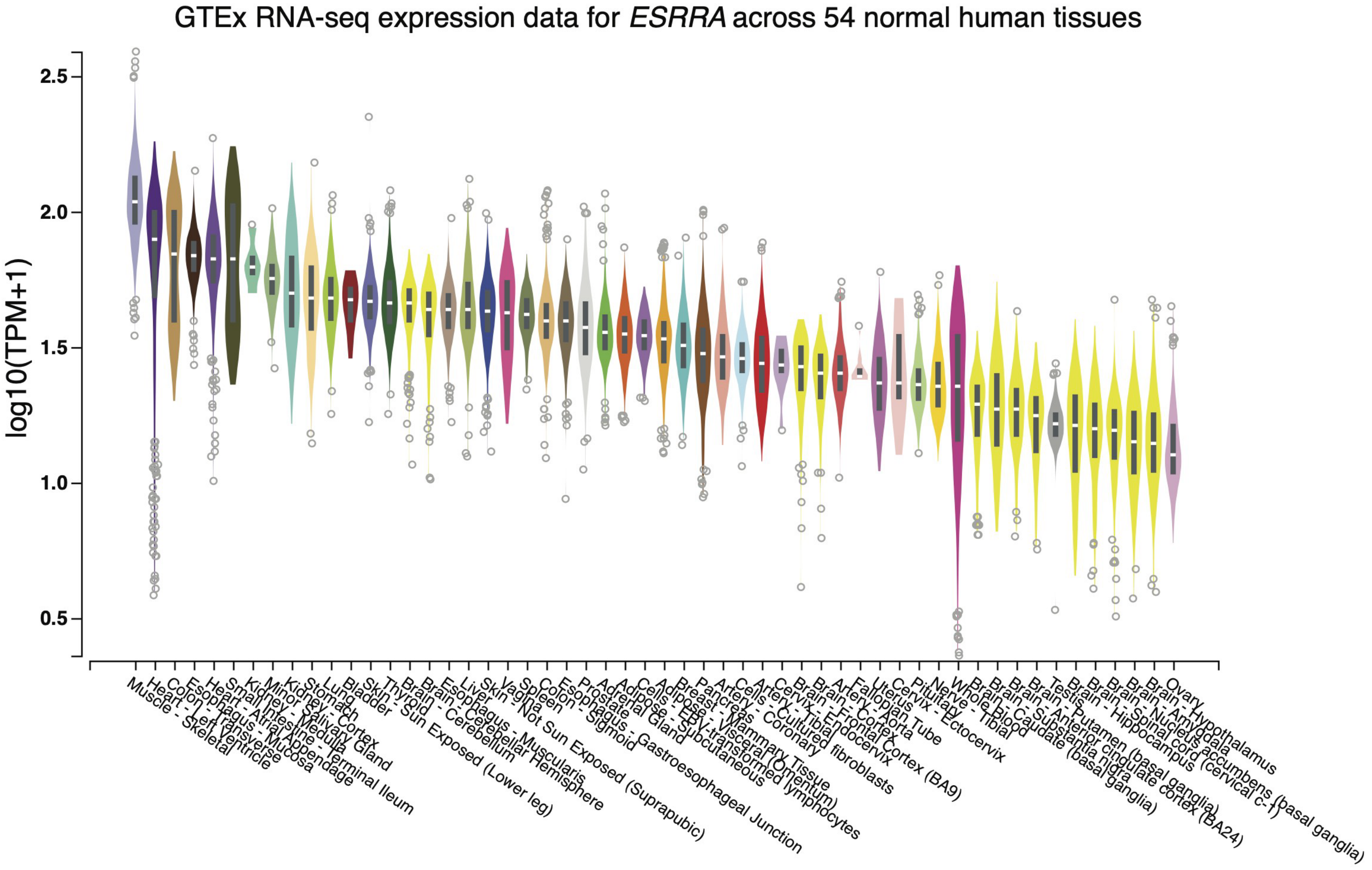

b

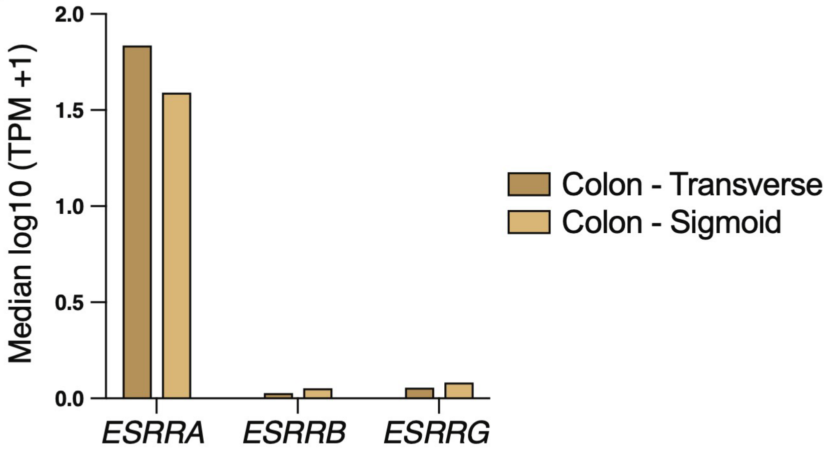

Supplemenntary Figure 1

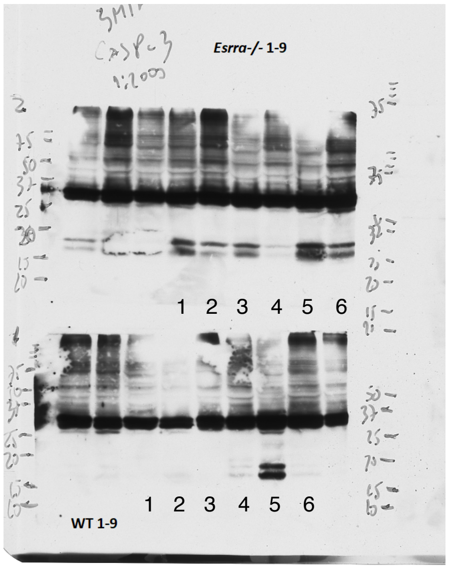

Caspase-3

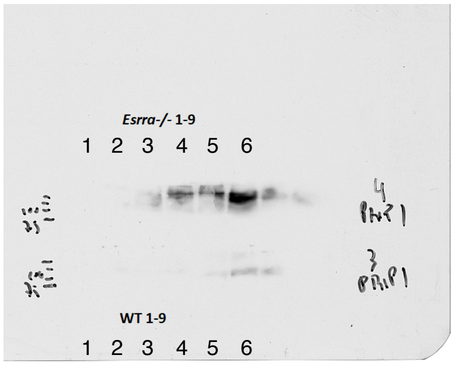

pRIPK1

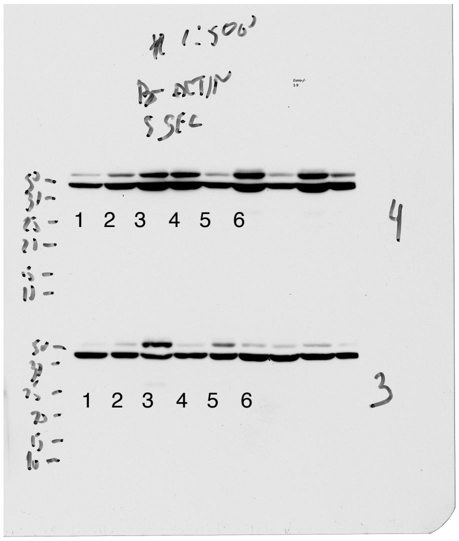

b-actin
(of the pRIPK1 blots)

Supplemenntary Figure 2
